# Metformin sensitizes lung cancer cells to treatment by the tyrosine kinase inhibitor erlotinib

**DOI:** 10.18632/oncotarget.22596

**Published:** 2017-11-21

**Authors:** Xiaofei Wang, Keqiang Chen, Ying Yu, Yi Xiang, Jae Hong Kim, Wanghua Gong, Jiaqiang Huang, Guochao Shi, Qingyun Li, Min Zhou, Thomas Sayers, Poonam Tewary, Beili Gao, Ji Ming Wang

**Affiliations:** ^1^ Department of Respiratory and Critical Care Medicine, Ruijin Hospital, Shanghai Jiaotong University School of Medicine, Shanghai 200025, China; ^2^ Cancer and Inflammation Program, Center for Cancer Research, National Cancer Institute, Frederick, MD 21702, USA; ^3^ Eye Institute, Affiliated Hospital of Nantong University, Nantong 226001, China; ^4^ Basic Research Program, Leidos Biomedical Research, Inc., Frederick, MD 21702, USA; ^5^ College of Life Sciences and Bioengineering, School of Sciences, Beijing Jiaoton University, Beijing 100044, China

**Keywords:** lung cancer, metformin, EGFR, erlotinib, phosphorylation

## Abstract

Lung cancer is one of the deadliest malignant tumors with limited treatment options. Although targeted therapy, using tyrosine-kinase inhibitors such as erlotinib (Erlo), has shown therapeutic benefit, only 15 % patients with mutated epidermal growth factor receptor (EGFR) in lung cancer cells are sensitive. Therefore, additional therapeutic strategy should be developed. In this study, we found that metformin (Met), which is widely used for the treatment of type 2 diabetes (T2D), sensitized lung cancer cells bearing wild-type EGFR to Erlo treatment by enriching cancer cells expressing higher levels of EGFR with persistent phosphorylation. As a consequence, combination of Met and Erlo more efficiently inhibited the growth of lung cancer cells both *in vitro* and in mice with xenografted tumors. Our results suggest a novel approach to treating lung cancer cases which are originally resistant to Erlo.

## INTRODUCTION

Lung cancer is one of the most common causes of cancer-related deaths worldwide and non-small cell lung cancer (NSCLC) accounts for 80% of all cases [1]. The incidence of lung cancer in the United States in 2016 alone is estimated at about 224,390 new cases and about 158,080 deaths (85,920 men and 72,160 women) [2].

In addition to traditional therapies including surgery, chemotherapy and radiation, the development of small-molecule protein kinase inhibitors, for example, tyrosine kinase inhibitors (TKIs), such as erlotinib (Erlo) that targets the ATP binding site of the intracellular domain of EGFR [3] has revolutionized the treatment of NSCLCs. EGFR mutations are significant predictors of treatment response to TKIs, but unfortunately, only 15 % of all lung cancers are expected to be sensitive [4].

EGFR (also known as ErbB-1 or HER1) belongs to the ErbB family of cell-surface receptor tyrosine kinases. In normal lung tissue, EGF triggers homodimerization of EGFR or heterodimerization with other ErbB members, leading to receptor phosphorylation and activation of downstream effectors such as ERK, PI3K and STAT3 [5], providing a robust signal for epithelial cell proliferation and survival. Such signal cascade fades away after normal organogenesis and tissue repair to maintain homeostasis [6]. However, dysregulated EGFR activation associated with receptor mutation, was found in the lungs with neoplastic and pre-neoplastic changes, including bronchial preneoplasia, the indolent bronchioalveolar carcinoma (BAC) and NSCLCs [6–8]. Thus, a desensitization mechanism to restrain or terminate EGFR activation has been disrupted during lung tumorigenesis. Therefore, aberrant EGFR activation becomes a target for the design of novel lung cancer treatment.

Metformin (Met), a prescription drug for type 2 diabetes (T2D), is an extensively studied metabolism regulator. Emerging evidence indicates the capacity of Met to decrease cancer risks in human [9]. Population-based studies demonstrated an association between Met use and improved survival among diabetic patients with cancers [10]. For example, diabetic lung cancer patients with proper glycemic control exhibited not only a better overall survival (OS) than those without proper glycemic control, but also a better OS even than patients without diabetes [11]. Met has therefore been used in clinical trials in cancer, extending to non-diabetic patients [10, 12]. However, the effect of Met, like other anti-cancer drugs, shows limitations. It is therefore important to consider combination of drugs to increase the therapeutic efficacy. In this study, we report the ability of Met to enrich human lung cancer cells expressing higher levels of wild type EGFR (EGFR^high^) and enhance their sensitivity to the therapeutic effect of the TKR inhibitor, Erlo.

## RESULTS

### Met reduces the proliferation of human lung cancer cells

**Figure 1:**
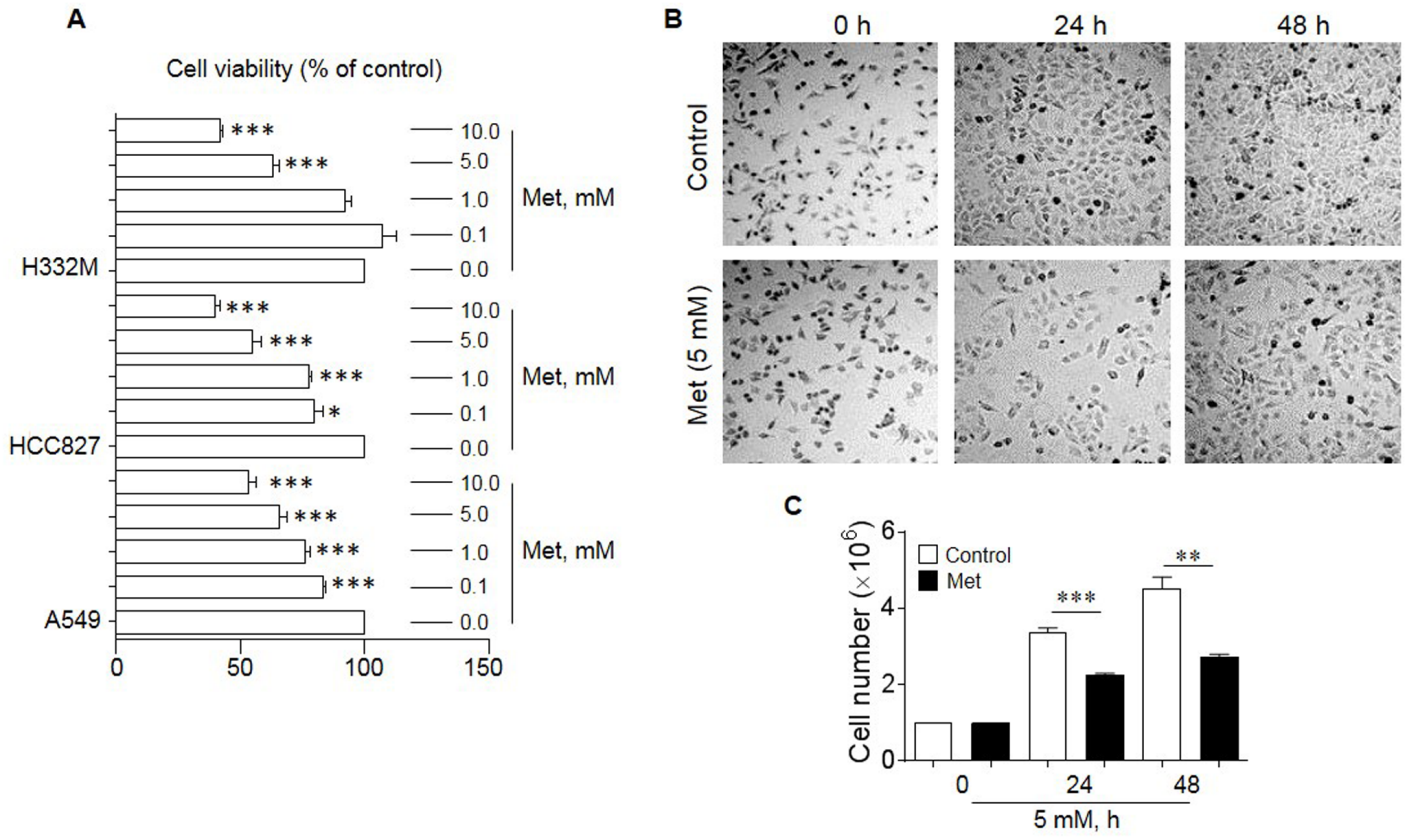
The effect of Met on the proliferation of human lung cancer cell lines Cell counting and MTT assays were performed to examine the proliferation of lung cancer cells in the presence or absence of different concentrations of Met for 24 and 48 h. **(A)** Suppression of the proliferation of human lung cancer cell lines (A549, HCC827 and H332M) by Met treatment for 48 h. Graphs represent the percentage of the cells in the presence of Met compared to cells cultured in the absence of Met (n = 3). ^*^ denotes significantly reduced cell number after Met treatment. ^*^ p < 0.05, ^***^p < 0.001. **(B)** Pictures of A549 cells cultured in the presence or absence of 5 mM Met for 24 and 48 h. **(C)** The mean number of A549 cells cultures in the presence or absence of 5 mM Met for 24 and 48 h. ^*^ denotes significantly decreased cell number after Met treatment as compared cells cultured in the absence of Met (Control). ^**^p < 0.01, ^***^p < 0.001.

Met has been shown to inhibit the proliferation of human cancer cell lines derived from the prostate, colon, gliomas and breast [13–15]. We therefore investigated the effect of Met on the *in vitro* growth of human lung cancer cell lines A549, HCC827 and H332M. As shown in Figure 1, Met dose-dependently inhibited the proliferation of all lung cancer cell lines tested with optimal inhibition at 5-10 mM after 48 h exposure (Figure 1A-C). We therefore choose 5 mM Met in subsequent experiments.

### Met induces the apoptosis of human lung cancer cells

**Figure 2:**
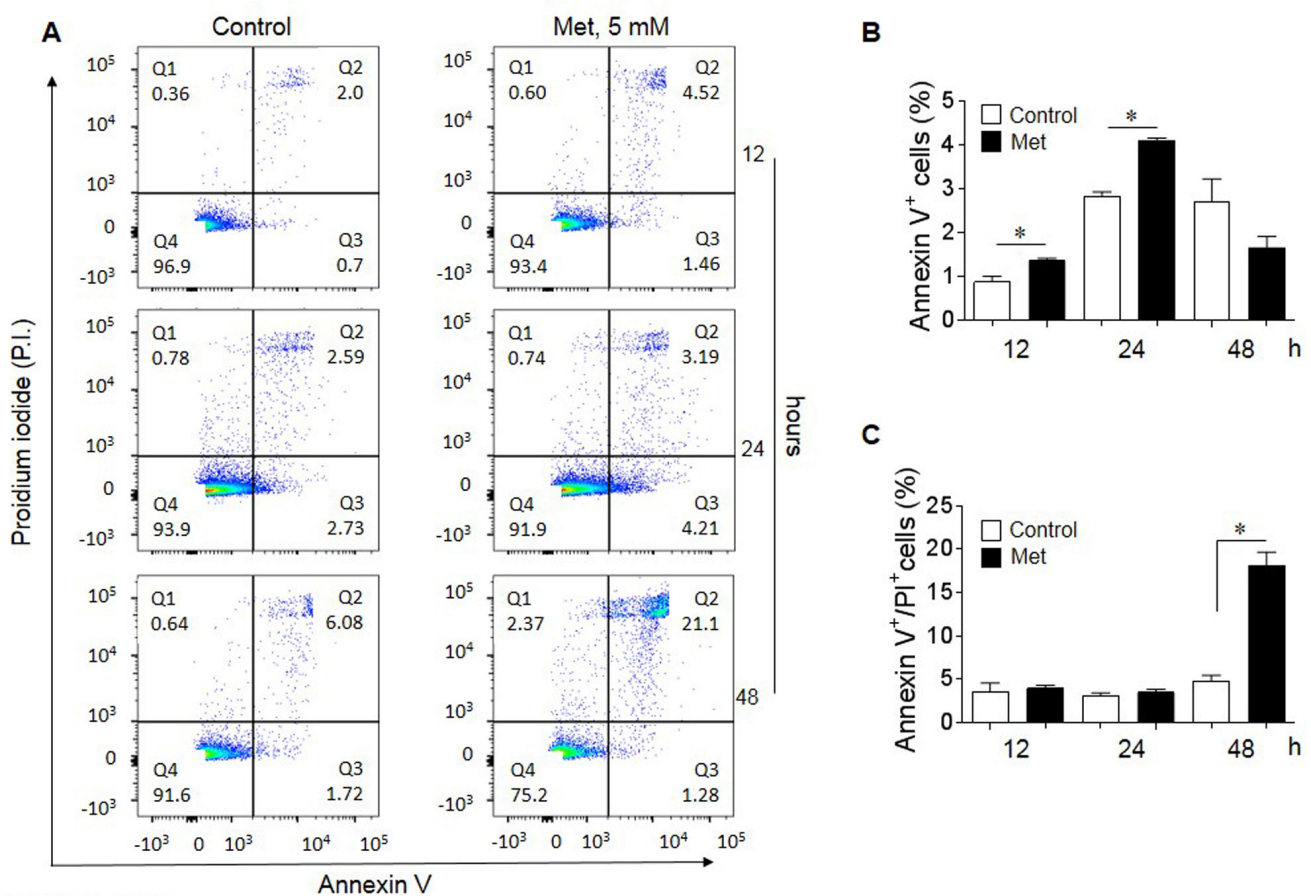
Induction of lung cancer cell apoptosis by Met Flow cytometry was performed to determine the pro-apoptotic effect of 5 mM Met on A549 lung cancer cells. **(A)** Apoptotic cells (%) following treatment with 5 mM Met for 12, 24 and 48 h. Quadrant (Q) 1 defines necrotic (PI single positive) cells; Q2 defines late apoptotic cells (annexin V and PI double positive); Q3 defines early apoptotic cells (annexin V single positive) and Q4 defines healthy cells (non-apoptotic cells). **(B)** Increased early apoptotic A549 cells after Met treatment for 12 and 24 h. Graphs represent the mean ± SEM of the percentage of apoptotic cells (n = 3). ^*^ denotes significantly increased percentage of early apoptotic cells after Met treatment compared to untreated cells (Control). ^*^p < 0.05. **(C)** The proportion of late apoptotic cells in the presence of absence of Met for 48 h. ^*^ Significantly increased number of late apoptotic cells after Met treatment compared to cells cultured in the absence of Met (Control). ^*^p < 0.05.

We next examined whether Met induced the apoptosis of human lung cancer cells. Figure 2 shows that Met at 5 mM induced early apoptosis of A549 lung cancer cells as stained with an anti-Annexin V antibody starting after 12 h of incubation (A-B). At 48 h of Met treatment, there was a significantly increased proportion of later apoptotic cells stained with propidium iodide (PI (Figure 2A-C). These results indicate that Met inhibits lung cancer cell proliferation by inducing apoptosis.

### Met sensitizes lung cancer cells to the cytotoxicity of Erlo

**Figure 3:**
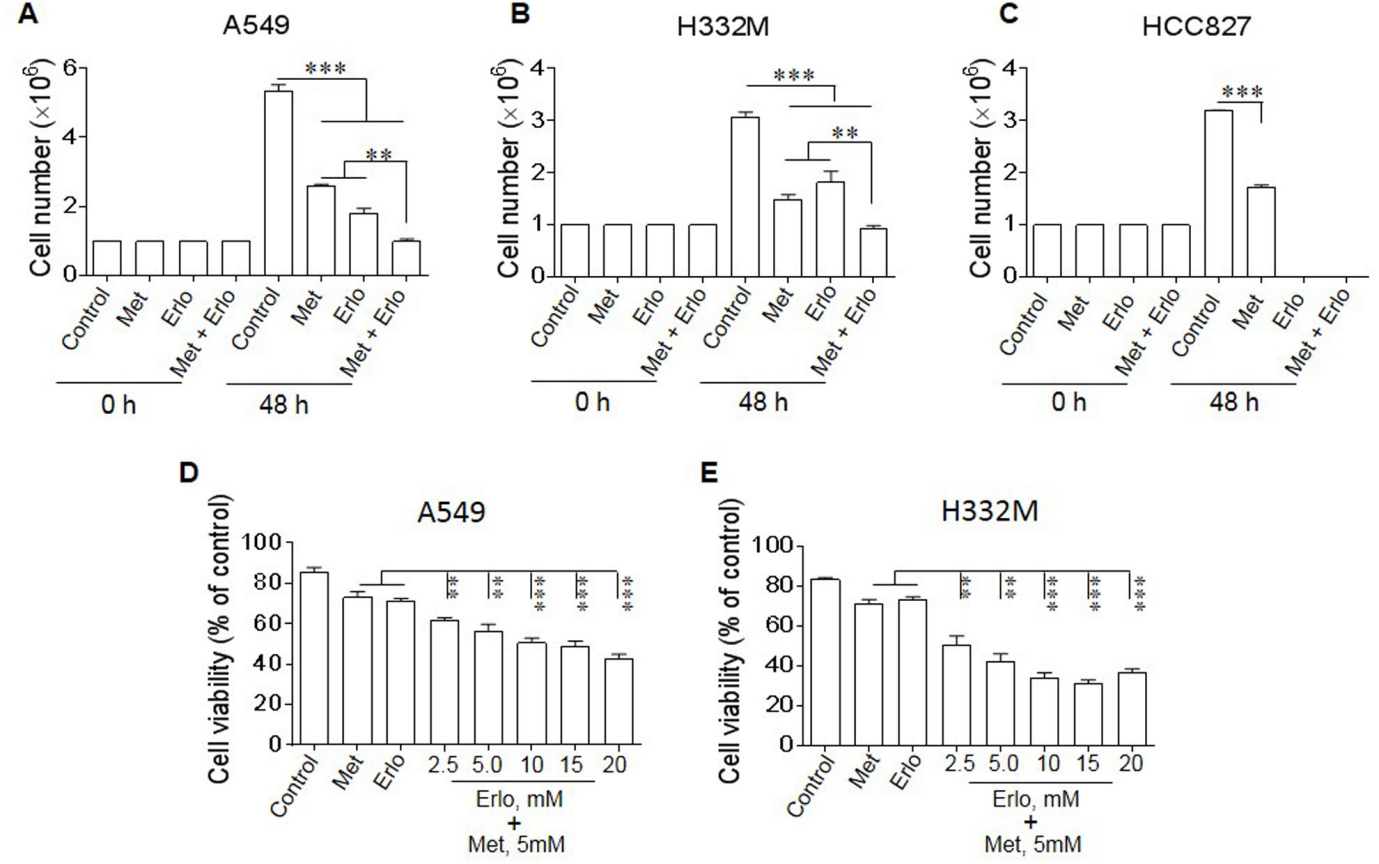
Sensitization of lung cancer cells to the cytotoxicity of Erlo by Met Lung cancer cells were cultured with Met (5 mM) in the presence or absence of Erlo (5 mM). The number of cells was counted after 48 h and the percentage of viable cells was calculated. Graphs represent the mean ± SEM of tumor cell numbers and the percentage of viable cells (n = 3). **(A-C)** The number of A549, H332M and HCC827 cells in the presence or absence of Met or Erlo alone or in combination. ^*^ denotes significantly decreased number of lung cancer cells cultured in the presence of both Met and Erlo compared the number of cells cultured in medium (Control) or in the presence of Erlo alone. ^**^ p < 0.01, ^***^ p < 0.001. **(D-E)** Inhibition of A549 and H332M cell proliferation by Met in the presence of different concentrations of Erlo calculated as the percentage in comparison with cells cultured in medium (Control) or with Met or Erlo alone. ^*^ denotes significantly decreased viability of the cells treated with Met in combination with Erlo compared to cells cultured in medium (Control) or with Met or Erlo alone. ^**^ p < 0.01, ^***^ p < 0.001.

Since at high doses, Met did not show further increased inhibition on lung cancer cell proliferation, we investigated whether the cells survived Met treatment remained sensitive to cytotoxicity of a receptor tyrosine-kinase inhibitor (TKI) erlotinib (Erlo) therefore benefit from a combined treatment. A549 and H332M human lung cancer cells are known to be resistant to TKIs because of the absence of mutations in EGFR on cell surface, whereas HCC827 human lung cancer cells contain mutated EGFR, thus are sensitive to TKIs. In fact, combination of Met and Erlo more potently inhibited the proliferation of A549 and H332M cell lines with wild type EGFR (EGFR WT) than Met or Erlo alone (Figure 3A-B). In contrast, Erlo alone was sufficient to maximally inhibit the proliferation of HCC827 cells with mutant EGFR (Figure 3C) and increasing Erlo concentration in combination with Met did not further enhance the effect of inhibited proliferation of A549 and H332M cell lines (Figure 3D-E). These results indicate that Met is able to increase the sensitivity of EGFR WT lung cancer cells to the toxicity of the TKI Erlo.

### Met enriches EGFR positive cells in A549 cells

**Figure 4:**
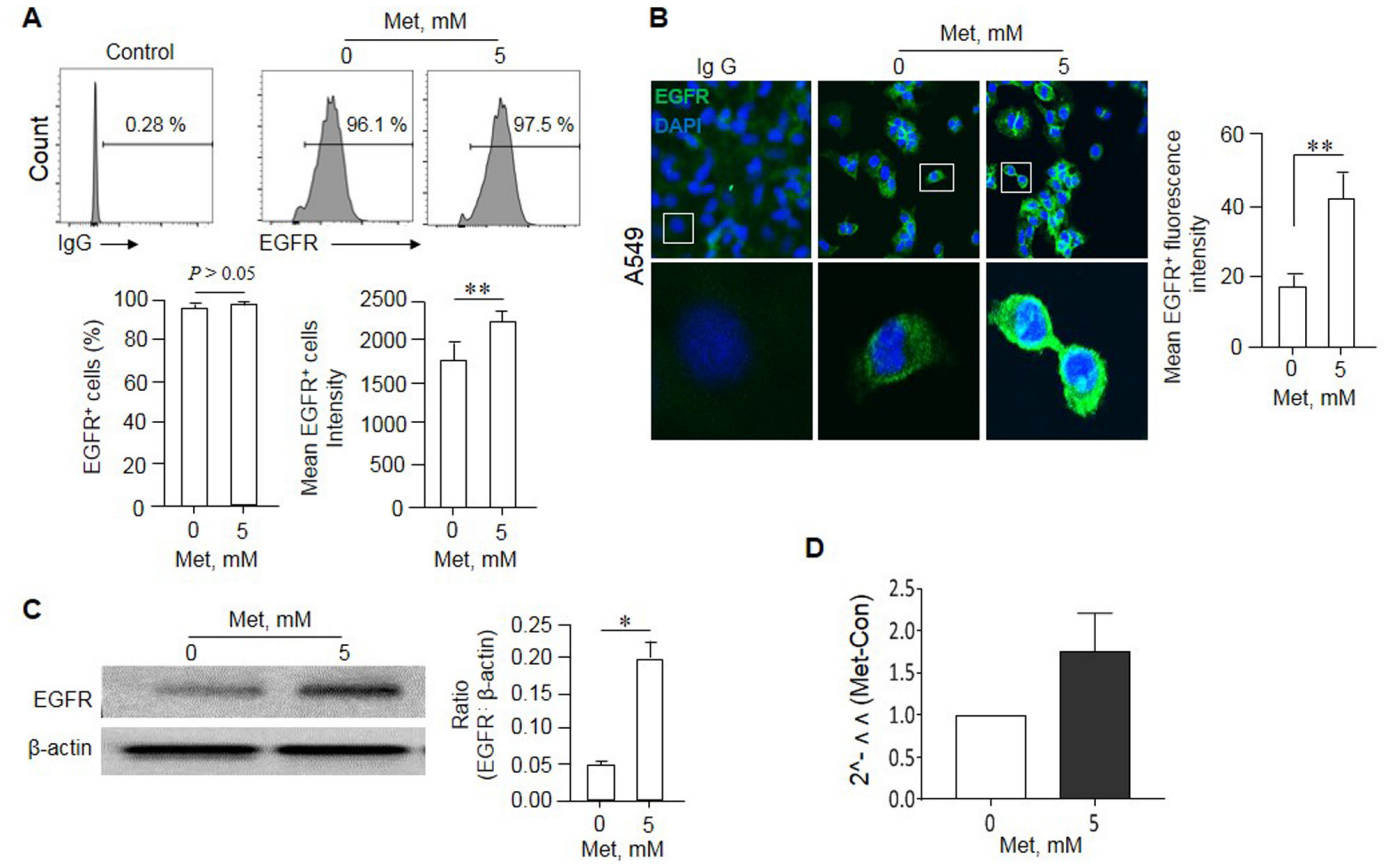
Enrichment of an EGFR^high^ population in A549 cells by Met A549 cells were cultured in the presence of Met (5 mM) for 48 h. The expression of EGFR by surviving A549 cells was examined by flow cytometry, Western blot and Immunofluorescence. **(A)** No significant difference in the percentage of EGFR positive cells in A549 cells line cultured in the presence of absence of Met. However, higher intensity of EGFR was shown by A549 cells surviving culture in the presence of Met. Graphs represent the mean ± SEM of the positivity and the intensity of EGFR fluorescence (n = 3). ^**^ Denote significantly increased (P < 0.01) intensity (P < 0.01) of EGFR fluorescence on A549 cells after culture with Met compared with the cells cultured in medium (Met 0). **(B)** Enhanced fluorescence of EGFR on A549 cells after culture with 5 mM Met for 48 h. ^**^ denote significantly increased (p < 0.01) fluorescence intensity on the surface of A549 cells after Met treatment as compared to cells cultures in medium (Met 0). **(C)** Higher EGFR protein expression by A549 cells cultured in the presence of Met (5 mM, 48 h) as measured by Western blot. Graphs represent the densitometry measurement of EGFR bands. ^*^ Denotes significantly increased EGFR protein expressed by Met treated A549 cells compared to cells treated with medium (Met 0). **(D)** Real time RT-PCR measurement of EGFR mRNA in A549 cells. No statistically significant difference was detected in EGFR mRNA expression levels between the cells cultured in the presence or absence of Met.

We next investigated the mechanisms why Met can sensitize human lung cancer cells bearing EGFR WT to the cytotoxicity of Erlo. We found that the remaining A549 cells after Met treatment expressed increased level of EGFR on the surface (EGFR^high^) as shown by more potent fluorescence intensity compared with the cells not treated with Met (Figure 4A-B). Western blot showed higher EGFR protein levels in A549 cell lysate after Met treatment (Figure 4C). Although the level of EGFR mRNA in A549 cells appeared to be also increased after Met treatment, repeated experiments did not reveal a statistically significant change (Figure 4D), suggesting the effect of Met occurs mainly at a post-transcriptional level.

### Met treatment results in persistent EGFR phosphorylation in A549 cells

**Figure 5:**
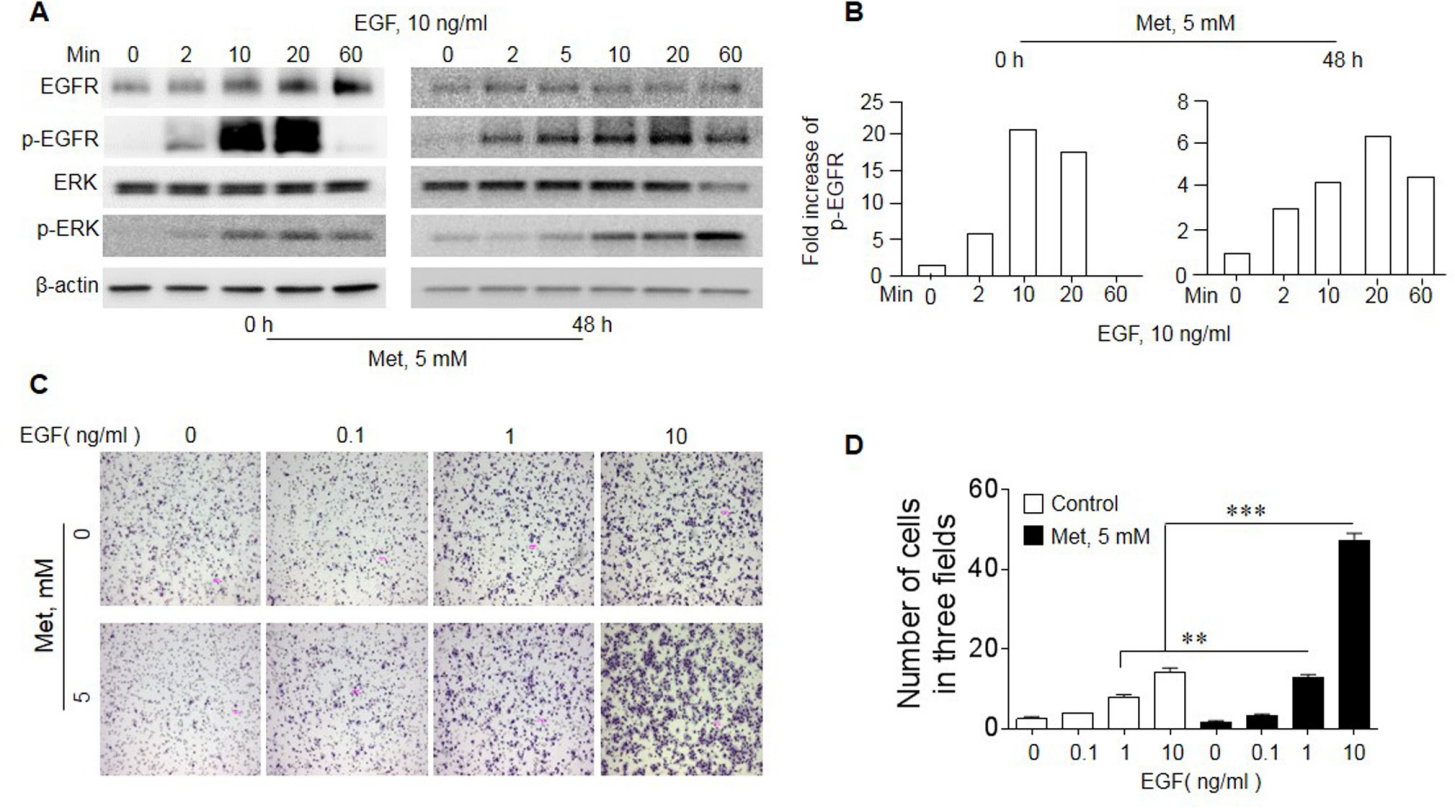
Increased sensitivity of EGFR in response to EGF in A549 cells cultured with Met A549 cells were cultured in the presence or absence of 5 mM Met for 48 h. The cells were then measured for responses to EGF stimulation. **(A)** The phosphorylation of EGFR and ERK in A549 cells. Cells cultured with Met were serum starved overnight before measurement of EGFR, p-EGFR (Y1068), Erk and p-Erk in response to EGF with Western blot. **(B)** Persistent EGFR phosphorylation (up to 60 min) in Met treated A549 cells in response to EGF (10 ng/ml). **(C)** Cell migration in response to EGF. A549 cells were cultured with or without Met (5 mM) for 48 h, followed by measurement of migration in response to different concentrations of EGF. **(D)** Graphs representing the mean ± SEM of the number of migrated A549 cells. ^*^ denotes significantly increased migration of Met treated A549 cells in response to EGF compared to cells cultured with medium (0). ^**^ p < 0.01, ^***^ P < 0.001.

We then investigated the mechanistic basis for Met to “sensitize” EGFR WT lung cancer cells to the inhibitory effect of Erlo. EGF-stimulated EGFR initiates a signaling cascade culminating in its transient phosphorylation and cell proliferation. In A549 cells, EGF induced EGFR phosphorylation with or without Met treatment (Figure 5A-B). In native A549 cells, the phosphorylation of EGFR was transient with rapid reduction in 20 minutes. However, Met pretreatment of the cells for 48 h resulted in persistent EGFR phosphorylation after EGF treatment, which lasted for more than 60 min (Figure 5A-B). Met pretreated A549 cells also showed more potent phosphorylation of ERK, a down-stream signaling molecules of EGFR signaling pathway, in response to EGF stimulation (Figure 5A). The increased signaling capacity of EGFR in Met-treated A549 cells was associated with more potent directional migration induced by EGF (Figure 5C-D). Thus, Met pretreatment enriches a population in A549 cells with increased biological function of EGFR WT, which becomes sensitive to the toxicity of Erlo.

### Met and Erlo additively inhibit the growth of xenograft tumors formed by A549 cells

**Figure 6:**
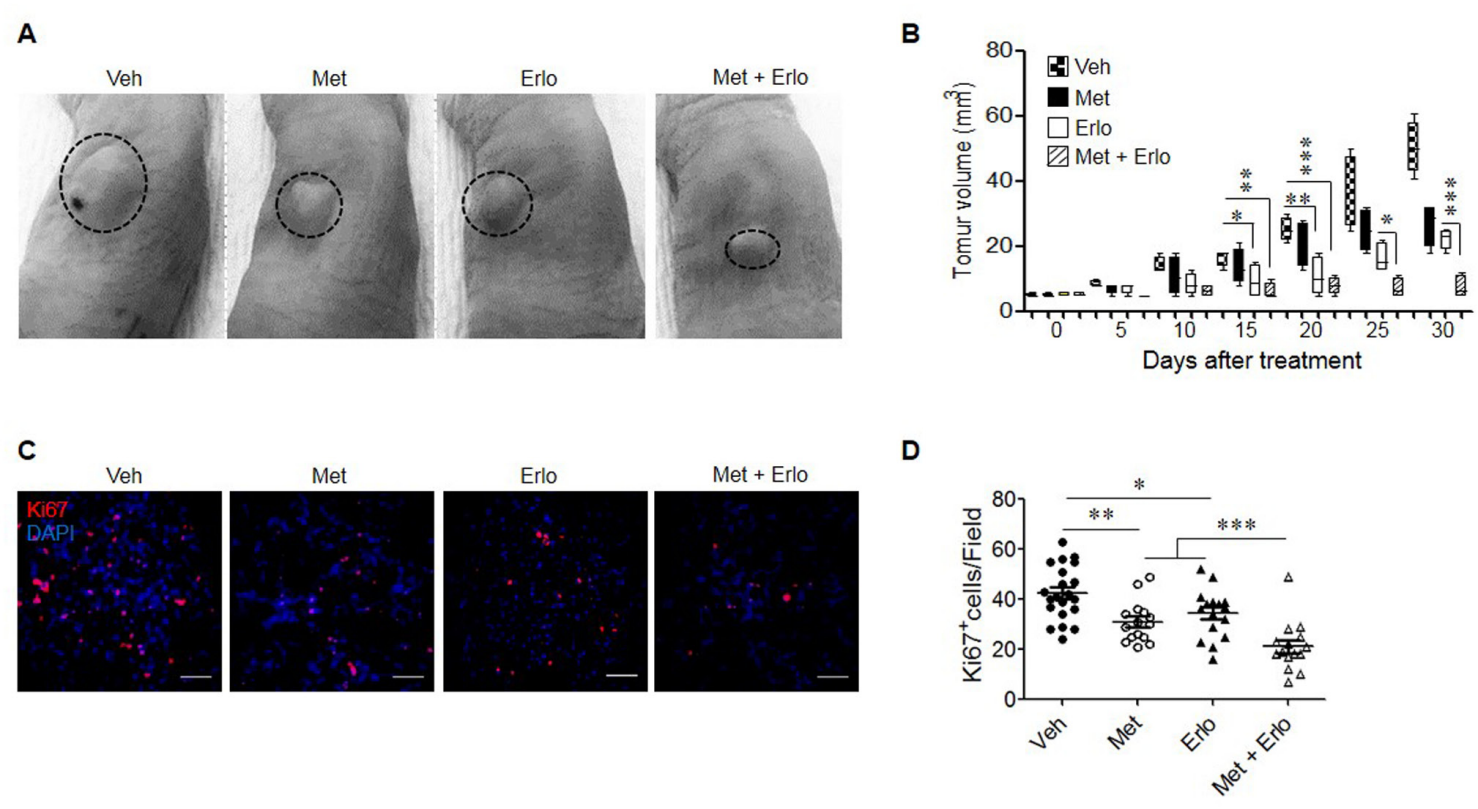
Enhancement of the therapeutic effect of Erlo on xenograft lung cancer by Met Human lung cancer cell line A549 (5 × 10^6^) in 200 μl PBS were subcutaneously injected into the left flank of nude mice. One week after implantation when tumors reached a volume of 4-6 mm^3^, mice were intraperitoneally injected with 250 mg/kg Met or 50 mg/kg Erlo or in combination, every 5 days for 5 cycles. The Erlo solvent Captisol (6%) was injected in control tumor bearing mice. At the indicated days post tumor cell implantation, tumor size was measured and at 30 days, mice were euthanized and tumors were harvested for histological examination. **(A)** Photographs of xenograft tumors at day 30 after A549 cell implantation. Veh: Captisol; Met: metformin; Erlo: erlotinib. **(B)** Growth kinetics of xenograft tumors in nude mice with or without treatment with Met or Erlo or in combination. ^*^ denotes significantly (P < 0.05) reduced size of tumors in mice treated with Met, Erlo, or in combination. ^**^ p < 0.01; ^***^ p < 0.001. **(C)** Ki67 staining for xenograft tumors at day 30 after A549 cell implantation, Red: Ki67, Blue: DAPI; Scale bar: 50 μm. **(D)** The Ki67^+^ cells from xenograft tumors at day 30 after A549 cell implantation were quantified. N = 15-21, 3-5 mice per group. ^*^ p < 0.05; ^**^ p < 0.01; ^***^ p < 0.001.

**Figure 7:**
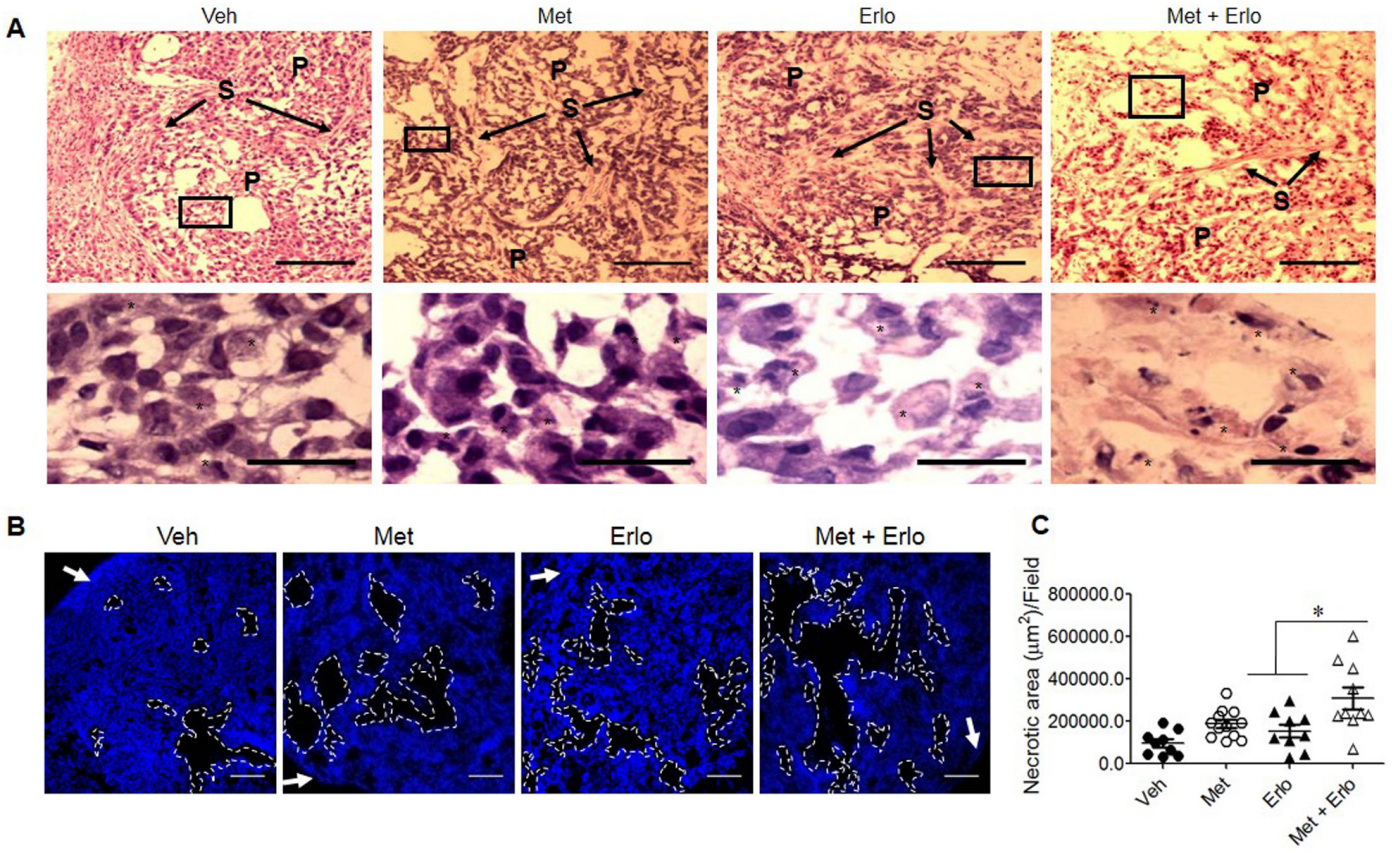
Enhancement of cell apoptosis by Erlo on xenograft lung cancer by Met **(A)** H&E staining for xenograft tumors at day 30 after A549 cell implantation. P: Parenchyma, S: Stroma. Star: Apoptotic-like cells. Up-panel, Scale bar: 100 μm; Down-panel, Scale bar: 20 μm. **(B)** Necrotic area in xenograft tumor tissues. White arrow: Capsule. Scale bar: 200 μm. **(C)** The necrotic areas in xenograft tumors at day 30 after A549 cell implantation were quantified. N = 9-12, 3-5 mice per group. ^*^ p < 0.05.

Based on the observations that Met and Erlo additively inhibited the growth of EGFR WT A549 lung cancer cells *in vitro*, we examined the therapeutic effect of these two agents on the growth of xenograft tumors in immuno-compromised mice. Figure 6A and 6B show that Met and Erlo alone administrated after tumor reaches 4-6 mm^3^ was able to partially inhibit the growth of tumors formed by A549 cells in nude mice. Combination of Met and Erlo exhibited a greater therapeutic effect on the growth of xenograft tumors than any single agent alone seen 30 days after tumor cell implantation. Then, we detected the expression of Ki67, a marker for cell proliferation, in the xenograft tumors formed by A549 cell line treated with Met and Erlo alone or combination. Figure 6C and 6D showed that Met and Erlo alone administrated was able to partially reduce the number of Ki67^+^ cells and combination of Met and Erlo exhibited a greater effect to diminish the Ki67^+^ cells in the xenograft tumor tissues than any single agent alone seen 30 days after tumor cell implantation. Histology analysis revealed that parenchyma was reduced, the nuclei of neoplastic cells were dense or disappeared and the arrangement of stromal cells was disorder in the tumor tissues treated with combination of Met and Erlo (Figure 7A). We also found that the necrotic areas were also increased in the tumor tissues treated with combination of Met and Erlo significantly as compared to that treated with any single agent alone (Figure 7B-C). These results demonstrate a combined therapeutic effect of Met and Erlo on xenograft tumors formed by EGFR WT human lung cancer cells.

### Enhancement of EGFR expression by Met and reduced phosphorylation of EGFR by combination Met and Erlo in the tumor cells from A549 cell line

**Figure 8:**
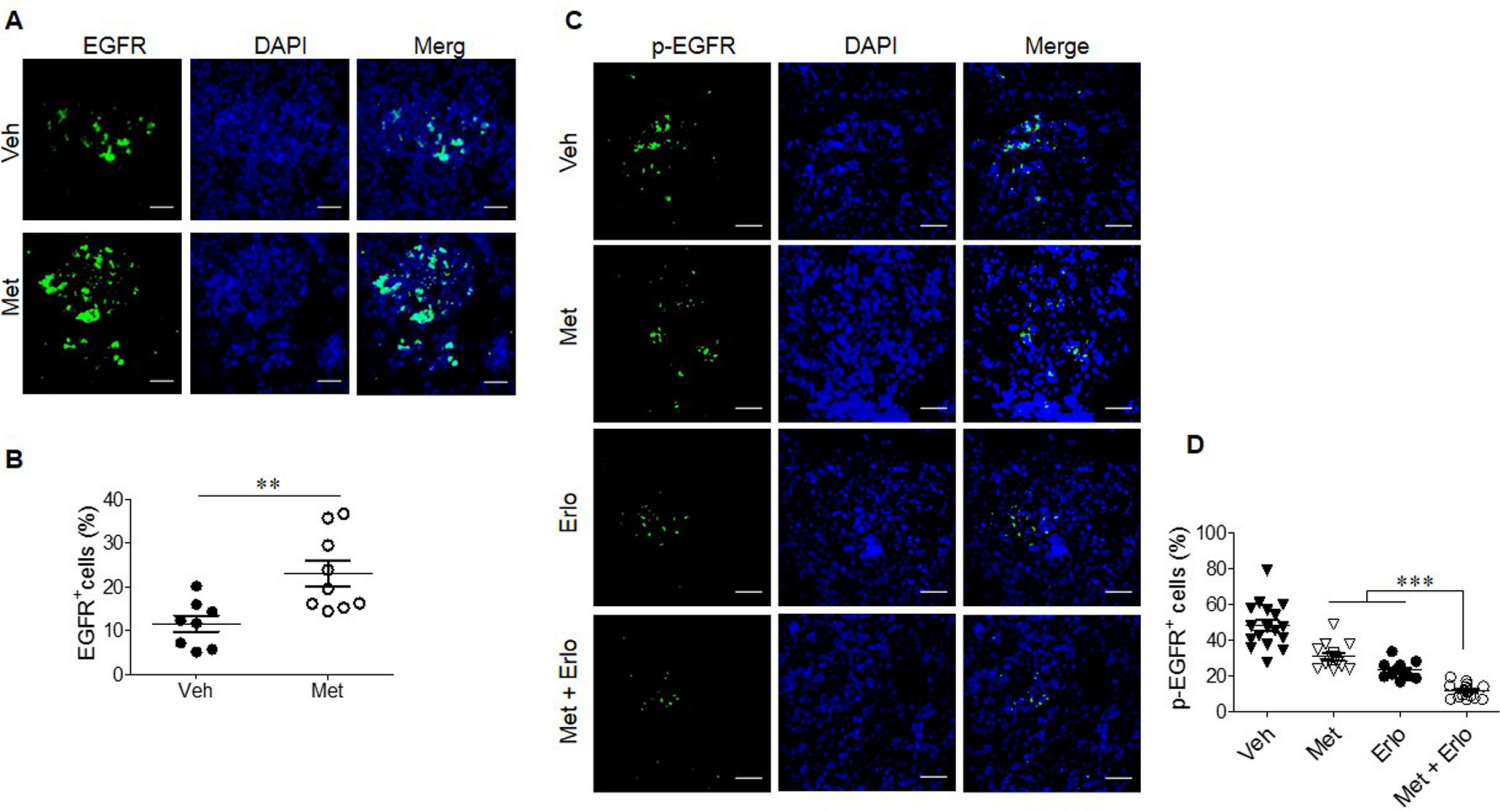
Enhancement of EGFR expression by Met and reduced phosphorylation of EGFR by combination Met and Erlo **(A)** Met promotes EGFR expression by xenograft tumor cells, Green: EGFR, Blue: DAPI; Scale bar: 50 μm. **(B)** The EGFR^+^ cells from xenograft tumors at day 30 after A549 cell implantation were quantified. The results indicated that the percent of EGFR^+^ cells in total cell number of each cancer cell nest, N = 12-14 cancer cell nests, 3-5 mice per group. ^***^ p < 0.001. **(C)** Reduced phosphorylation of EGFR in xenograft tumors at day 30 after A549 cell implantation treated with combination Met and Erlo, Green: p-EGFR, Blue: DAPI; Scale bar: 50 μm. **(D)** The cells expressing p-EGFR in xenograft tumors at day 30 after A549 cell implantation were quantified. The results indicated that the percent of p-EGFR^+^ cells in total cell number of each cancer cell nest, N = 12-15 cancer cell nests, 3-5 mice per group. ^***^ p < 0.00.

We further investigated the mechanism of the combined therapeutic effect of Met and Erlo on xenograft tumors. Figure 8A-B revealed that Met treatment up-regulated EGFR expression in the tumor cells significantly as compared to control tumor cells. Combination of Met and Erlo exhibited a greater effect to inhibit phosphorylation of EGFR significantly than Erlo or Met alone in the xenograft tumor tissues seen 30 days after tumor cell implantation (Figure 8C-D). Put together, these results demonstrate that Met, which is widely used for the treatment of type 2 diabetes (T2D), sensitized lung cancer cells bearing wild-type EGFR to Erlo treatment by enriching cancer cells expressing higher levels of EGFR with persistent phosphorylation.

## DISCUSSION

Met has been used to treat T2D for nearly 60 years. Met reduces circulating glucose and insulin levels by inhibiting gluconeogenesis in the liver. This is due to the ability of Met to indirectly activate AMP-activated protein kinase (AMPK) by inhibiting oxidative phosphorylation in liver cells [16]. Recently Met class drugs have been shown to possess anticancer properties [17–19], while the mechanisms were controversial. Recent research demonstrated that Met inhibits cell growth by attenuating mitochondrial respiratory capacity, which restrains the transit of RagA-RagC GTPase heterodimer through nuclear pore complex (NPC). Nuclear exclusion renders RagC incapable of gaining GDP-bound state necessary for stimulating rapamycin complex-1 (mTORC1) in mammalian cells. Met-induced inactivation of mTORC1 subsequently inhibits cell growth through transcriptional induction of acyl-CoA dehydrogenase family member-10 (ACAD10) [20].

This pathway enables Met to kill cancer cells thus illuminating potential application in cancer treatment. Epidemiologic and preclinical evidence also has inspired the adoption of combination therapy, in which Met inhibits the growth of tumor initiating cells in breast cancer cell lines and prevents the relapse of tumors *in vivo* when combining with chemotherapy [21]. Our *in vitro* experiments showed that Met treatment resulted in the selection of A549 lung cancer cells that expressed increased levels of wild type EGFR on the cell surface (EGFR^high^). These enriched cells showed more persistent responses to EGF in EGFR phosphorylation. These cells also became more sensitive to the TK inhibitor Erlo as shown by reduction in proliferation and more importantly, reduced growth of xenograft tumors formed by such cells. Therefore, combination of Met and Erlo exhibited a greater therapeutic effect on the growth of xenograft tumors than any single agent alone administered after tumors reached a size of 4-6 mm^3^.

Previous study also revealed that Met in combination with first-generation TKI effectively increased the sensitivity of TKI-resistant lung cancer cells and blocked tumor growth in xenografts, associated with decreased IL-6 secretion, reversal of EMT and dampened IL-6/STAT3 signaling [22]. Preclinical studies indicated that Met inhibits PI3K/AKT/mTOR signaling and IGF-1R [23–25], and combination of Met with gefitinib markedly reduces the proliferation of NSCLC cell-lines harboring the wild-type LKB1 gene by inducing LKB1-mediated activation of AMPK, which in turn inhibits mTOR signaling [26]. Our study suggests a pathway differs from mTOR cascade in that EGFR^high^ lung cancer cells survive Met with sustained phosphorylation of EGFR in response to EGF, and increased sensitivity to Erlo, one of the most wildly used EGFR-TKIs at present in the clinic.

Erlo binds the ATP binding pocket in EGFR and inhibits the phosphorylation of EGFR, and its activation [27]. In NSCLC cell line HCC827 expressing mutated EGFR, which is extremely sensitive to Erlo, Met did not show an additive inhibition on cell growth. Thus, our findings are consistent with the report that Met overcame resistance to Erlo in lung cancer cells [22]. In support of this, a retrospective study reports that Met may delay the onset of acquired resistance to EGFR-TKI in NSCLC patients with T2D [28]. Further research is warranted to elucidate the precise mechanisms for the capacity of Met to enrich EGFR WT lung cancer cells and to enhance the signaling potential of EGFR WT thus more sensitive to the inhibitory effect.

In conclusion, our study provides novel evidence that Met sensitizes NSCLC cells with EGFR WT to Erlo. Since Met and Erlo are well-tolerated after oral administration, our current results may readily be translated into clinical trials and to serve as a platform for targeted therapy of TKI resistant lung cancer.

## MATERIALS AND METHODS

### Reagents

Erlo (s1023) was purchased from Selleckchem (Houston, TX); Met (1396309) was from Sigma-Aldrich (St. Louis, MO). Recombinant human-EGF was purchased from Sigma-Aldrich. Antibodies against EGFR and phosphor (p)-EGFR (Y1068), p-ERK1/2 and ERK1/2, GAPDH and horseradish peroxidase (HRP)-linked anti-rabbit IgG antibody for Western blotting were purchased from Cell Signaling Technology (Beverly, MA). EGFR antibody used for flow cytometry was purchased from BD Biosciences (San Jose, CA). CellTiter 96^®^ non-radioactive cell proliferation assay kit was purchased from Promega (Madison, WI). Apoptosis Detection kit (FITC Annexin V) was also purchased from Cell Signaling Technology. Dimethyl Sulfoxide (DMSO) used for dissolving Erlo experiments was from Sigma-Aldrich. Captisol used for dissolving Erlo for *in vivo* injection was purchased from Captisol^®^ (San Diego, CA).

### Cell lines and culture

Human lung cancer cell lines A549, H332M and HCC827were obtained from American Type Culture Collection (Manassas, VA) and maintained in National Cancer Institute DCTD Tumor Repository. All three cell lines were cultured in RPMI 1640 containing 10% FCS.

### Animals

Female nude mice (Athymic Ncr-nu/nu) were purchased from Charles River Laboratories Inc (Frederick, MD). All mice were housed in the animal facility at Frederick National Laboratory for Cancer Research (Frederick, MD) and were used at the age of 10 weeks. Animal care was provided in accordance with procedures outlined in the Guide for Care and Use of Laboratory Animals (National Research Council, 1996, National Academy Press, Washington D.C.).

### Quantitative-PCR

Total RNA was extracted from human lung cancer cells with an RNeasy mini kit and depleted of contaminating DNA with RNase-free DNase (Qiagen, Valencia, CA). The first strand cDNA was synthesized with the Transcriptor First Strand cDNA Synthesis Kit (Roche Diagnostics, IN). The primers for human EGFR were: forward, 5’-AGGTGGTCCTTGGGAATTTG and reverse, 5’-ACTGTGTTGAGGGCAATGAG; β-actin primers were: forward, 5’-TGCGTGACATTAAGGAGAAGC and reverse: 5’-GGAAGGAAGGCTGGAAGAGTG.

### Chemotaxis

Chemotaxis assays were performed in 48-well chemotaxis chambers (NeuroProbe, Gaithersburg, MD). The upper and lower compartments of the chambers were separated by a 10 μm pore-sized polycarbonate filter (GE Osmonics Labstore, Minnetonka, MN) coated with 50 μg/ml collagen type I (BD Biosciences, San Jose, CA). A 28-30 μl aliquot of chemoattractant was placed in the wells of the lower compartment, and 50 μl of human lung cancer cells (each at 1 × 10^6^ cells per ml of RPMI 1640 medium containing 1% BSA and 25 mM HEPES) were placed in the wells of the upper compartment. After 4 h incubation at 37°C, the filters were collected, removed of non-migrating cells, rinsed with PBS, fixed and stained with Three-Step solutions (Richard-Allan Scientific, Kalamazoo, MI). Migrated cells were counted in 5 random fields at 400 magnifications under light microscopy. The results were expressed as the mean ± SEM of migrated cells or if applicable, as chemotaxis index (CI) representing fold increase in tumor cells migrated in response to chemoattractants over the baseline migration in the absence of chemoattractants (to control medium).

### Western blot

Tumor cells were lysed with lysis buffer (20 mM Tris, pH 7.5), 1 mM EDTA, 150 mM NaCl, 1 mM EGTA, 1 mM β–glycerophosphate, 1% Triton X-100, 2.5 mM sodium pyrophosphate, 1 mM Na3VO4, 4 μg/ml aprotinin, 4 μg/ml leupeptin, 4 μg/ml pepstatin, and 1 mM PMSF). Protein samples were separated by 10% SDS-PAGE electrophoresis and transferred to nitrocellulose membranes. Nonspecific protein binding was blocked with 5% nonfat dried milk in Tris-buffer saline containing 0.1% Tween 20 (TBS-T) at room temperature (RT) with agitation. The nitrocellulose membranes were incubated with primary antibodies for overnight at 4°C, rinsed with TBS/TBS-T, and subsequently incubated with HRP-conjugated secondary antibodies for 1 h at room temperature (RT). Images were quantified using the Image J 1.4.3.67 (NIH software). The protein content was normalized to the level of β-actin.

### Flow cytometry

Tumor cells plated in flasks (1.0 × 10^6^) were co-cultured with Met in the presence or absence of Erlo for different time periods. The cells were then stained with Annexin V (FITC conjugated) and Propidium Iodide (PI) according to the manufacturer’s instructions. Apoptosis was detected by flow cytometry (BD LSR II, San Jose, CA).

### Immunofluorescence for lung cancer cells

For staining of EGFR, lung cancer cells were seeded at 2.0 × 10^4^ cells/well on 8-well chamber slides (NalgeNunc International Co., Naperville, IL) for 48 h. The cells were then fixed in 2% paraformaldehyde for 20 min at RT, washed with PBS 3 times for 5 min each, and incubated with 5% normal goat serum (Sigma-Aldrich, St. Louis, MO) in PBS plus 0.05% Tween 20 for 1 h to block nonspecific antibody binding. The samples were then incubated with anti-human EGFR antibody at 1:50 dilution for 2 h at RT followed with PE-conjugated goat anti-rabbit IgG (BD Biosciences) in PBS containing 1% BSA for 60 min. After staining with DAPI to visualize nuclei, the samples were analyzed under a laser-scanning confocal fluorescence microscope (ZeissLSM510 NLO Meta). The intensity of green fluorescence detected for EGFR was analyzed with Image J 1.4.3.67.

### Lung cancer cell proliferation

Lung cancer cell proliferation was examined MTT (3-(4,5-Dimethylthiazol-2-yl)-2,5-Diphenyltetrazolium Bromide) assays. Briefly, cancer cells were cultured in 96-well plates at 5 × 10^4^ cells per well in RPMI 1640 with 10% FCS in the presence of Met or Erlo or in combination. After 48 h, the cells were measured for MTT uptake at absorbance 570 nm with a microplate reader. The results were expressed as the mean ± SE of OD values.

### Tumor implantation

Human lung cancer cell line A549 (5 × 10^6^) in 200 μl PBS were subcutaneously injected into the left flank of nude mice. The weight of mice and the tumor size were monitored every other day and tumor volume was calculated as follows: Volume (mm^3^) = length × width × width × 0.52 [29, 30]. One week after implantation (when tumors reached a volume of 4-6 mm^3^), mice (4 mice per group) were intraperitoneally (i.p.) injected with 250 mg/kg Met or 50 mg/kg Erlo or in combination, every 5 days for 5 cycles. PBS and the Erlo solvent (6% Captisol) were injected in control tumor bearing mice. At the indicated days post tumor cell implantation, mice were euthanized and tumors were harvested for histological examination.

### Histology and immunofluorescence for xenograft tumors

Histological analyses were performed on fresh-frozen, optimal cutting temperature (OCT) compound-embedded xenograft tumors formed by human lung cancer cell line A549. The tumor tissues were sectioned for 5 (for H&E staining) or 10 mm (for Ki67, EGFR, p-EGFR staining). Slides were fixed in 4% neutral buffered formalin for 8 min. Haematoxylin and eosin (H&E) was performed. Immunofluorescence analyses were performed on fresh-frozen, OCT-embedded tumor sections (10 mm). Slides fixed in 4% neutral buffered formalin for 8 min were stained with anti-human EGFR, p-EGFR antibodies (Cell signaling, 1/50 dilution) followed by a biotinylated anti-rabbit Ig secondary antibody (BD Biosciences, San Jose, CA, 1/100 dilution) and then Streptavidin-FITC (eBioscience, 1/100 dilution). 4, 6-Diamidino-2-phenylindole (DAPI) was used to stain the nucleus.

### Statistical analysis

All experiments were performed at least three times. Representative and reproducible results were shown. Statistical analysis was performed with Prism Version 6.0 software (GraphPad Software, La Jolla, CA). All statistical tests were two-sided. The significance of the differences was assessed by Student’s *t* test. *P* < 0.05 was considered as statistically significant.

## Abbreviations

Erlo: erlotinib
EGFR: epidermal growth factor receptor
Met: metformin
NSCLC: non-small cell lung cancer
TKIs: kyrosine kinase inhibitors
AMPK: AMP-activated protein kinase
